# Initial active surveillance for patients with metastatic renal cell carcinoma: 10 years' experience at a regional cancer Centre

**DOI:** 10.1002/cam4.5330

**Published:** 2022-10-07

**Authors:** Mark Stares, Vishwani Chauhan, Jigi Moudgil‐Joshi, Qiu G. Kong, Jahangeer Malik, Aravindhan Sundaramurthy, Tony Elliott, Edward Mains, Steve Leung, Alexander Laird, Stefan N. Symeonides

**Affiliations:** ^1^ Edinburgh Cancer Centre Western General Hospital, NHS Lothian Edinburgh UK; ^2^ Institute of Genetics and Cancer Cancer Research UK Edinburgh Centre, University of Edinburgh Edinburgh UK; ^3^ Department of Urology Western General Hospital, NHS Lothian Edinburgh UK

**Keywords:** active surveillance, c‐reactive protein, prognosis, renal cell carcinoma, systemic anticancer therapy

## Abstract

A subset of patients with metastatic renal cell carcinoma (mRCC) follow an indolent disease course and may benefit from initial active surveillance (AS). However, selecting patients suitable for this approach is challenging. To investigate this we sought to define outcomes of patients with mRCC suitable for initial AS. All patients with mRCC clinically selected for initial AS at the Edinburgh Cancer Centre between January 2010 and December 2020 were identified. Key inflammatory biomarkers (haemoglobin, white cell count, neutrophil count, platelets, C‐reactive protein [CRP], albumin, corrected calcium) and the International Metastatic RCC Database Consortium (IMDC) risk score were measured. The relationship between these and time to systemic anticancer therapy (tSACT) and overall survival (OS) was analysed. Data were available for 160 patients. Estimated median overall survival was 88.0 (interquartile range [IQR] 34.0–127.0) months. Median tSACT was 31.8 (IQR 12.0–76.3) months. On multivariate analysis, only CRP was predictive of tSACT (HR 2.47 [95% CI:1.59–3.85] *p* < 0.001) and OS (HR 3.89 [95% CI:2.15–6.83] *p* < 0.001). Patients with CRP > 10 mg/L were more likely to commence SACT within 1 year than those with CRP≤10 mg/L (41% vs. 18%, Relative Risk 2.16 (95% CI:1.18–3.96) (*p* = 0.012)). IMDC risk score was not predictive of tSACT or OS. Active surveillance is an appropriate initial management option for selected patients with mRCC. CRP, a biomarker of systemic inflammation, may provide additional objective information to assist clinical decision‐making in patients with mRCC being considered for initial AS. Although this is a retrospective observational study, the cohort is well defined and includes all patients managed with initial AS in an inclusive real‐world setting.

## Graphical abstract

Despite being recommended by clinical guidelines, selecting patients with metastatic renal cell carcinoma for initial active surveillance remains challenging. We present the largest cohort of such patients, adding to the growing body of evidence that AS is an appropriate initial management approach for selected patients. For the first time, we demonstrate that C‐reactive protein, a biomarker of systemic inflammation, predicts those who benefit most from this approach.

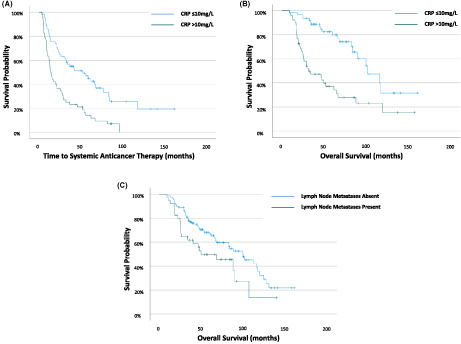

## 1 INTRODUCTION

Metastatic renal cell carcinoma (mRCC) is a heterogenous condition with variable biology and clinical outcomes. Incurable, with limited prognosis, systemic anticancer therapies (SACT) are the mainstay of treatment for most patients. Immune checkpoint inhibitor (ICI) immunotherapy agents, either as doublet‐therapy or in combination with a vascular endothelial growth factor receptor (VEGFR) inhibitor, have largely superseded the longstanding use of single agent VEGFR‐inhibitor therapy in the first‐line setting.^1^, ^2^ The prospect of clinical benefit from SACT must be balanced by the need for ongoing therapy and the risk of adverse events, some of which are long‐lasting or potentially life threatening. This is particularly pertinent in real‐world clinical settings where patients may be less fit and more co‐morbid than those enrolled in clinical trials.

A subset of patients with mRCC follow an indolent disease‐course characterised by slowly progressive growth of low‐volume, asymptomatic metastatic disease.^3^, ^4^, ^5^ These patients may benefit from delayed initiation of SACT by utilising an initial active surveillance (AS) approach. There is a growing body of evidence to suggest this approach is safe, preserves quality of life and does not adversely impact on prognosis.^4^, ^5^, ^6^, ^7^ Existing guidelines, whilst recommending that initial AS should be considered in some patients, remain elusive about how this cohort is identified, recommending AS in patients with “limited tumour burden and few symptoms”.^8^, ^9^ Patients who are clinically selected for initial AS are of better Eastern Cooperative Oncology Group performance status (PS), more favourable International Metastatic RCC Database Consortium (IMDC) risk score and have fewer metastatic disease sites than those who immediately commence SACT.^5^ In patients undergoing initial AS, higher number of IMDC adverse risk factors and higher numbers of metastatic disease sites are associated with a shorter surveillance period.^4^ However, these observations have had limited formal validation.^8^ Novel objective biomarkers to assist clinical decision‐making in patients with mRCC being considered for initial AS would be valuable tools.

The IMDC risk score, originally developed to predict survival in patients with mRCC treated with VEGFR‐inhibitors, is now used in multiple clinical settings in RCC.^2^, ^8^, ^10^ Three component factors of the IMDC score (haemoglobin (Hb), neutrophil count (NC), platelets) are considered biomarkers of the systemic inflammatory response. Along with other individual blood biomarkers (i.e., white cell count (WCC), albumin, C‐reactive protein (CRP) and lactate dehydrogenase (LDH) etc) or composite scores, such as the modified Glasgow Prognostic Score (mGPS), these inflammatory biomarkers are firmly established as having prognostic value in patients with cancer.^11^, ^12^, ^13^, ^14^

The Edinburgh Cancer Centre (ECC) serves a population of approximately 1.5 million across the Southeast of Scotland, UK. Utilising real‐world clinical data, we characterise patients with mRCC undergoing AS and identify objective biomarkers to select patients for this treatment strategy.

## 2 MATERIALS & METHODS

### 2.1 Study population

All patients with mRCC managed with initial AS at the ECC between January, 2010 and December, 2020 were identified from a comprehensive clinical database. Eligible patients were ≥ 18 years and had a diagnosis of metastatic RCC. All cases underwent clinicopathological assessment by a specialist renal cancer multidisciplinary team. All patients were reviewed in the specialist oncology clinic, were clinically suitable to receive SACT and had commenced on AS prior to receiving any SACT. The decision to start AS was made at the clinicians discretion, in consultation with the patient, based on published guidelines recommending initial AS in patients with limited tumour burden and few symptoms.^8^, ^9^ Patients who had received any prior adjuvant/neoadjuvant or metastatic systemic anticancer therapy were excluded.

### 2.2 Procedures and assessments

Patient demographics, clinical and pathological data were recorded. Biomarkers of the systemic inflammatory response (Hb, WCC, NC, platelets, albumin, corrected calcium [c.Ca2+] and CRP) at the time of diagnosis of metastatic disease were recorded. These factors were categorised within normal limits, in line with previous work in this area.^11^, ^12^, ^15^, ^16^ The mGPS and IMDC were calculated.

All data were collected as part of routine oncology work up in keeping with standard of care. No patient identifiable data were used. The presented work was in accordance with guidelines from ACCORD (Academic and Clinical Central Office for Research and Development, NHS Lothian and University of Edinburgh) and study‐specific consent was not required.

### 2.3 Statistical analysis

Time to SACT (tSACT), defined as the number of months from the date of first radiological finding of mRCC to starting SACT or death, or censorship if SACT had not commenced at follow‐up date (January 2, 2022), was calculated. Overall survival (OS), defined as the number of months from the date of first radiological finding of mRCC to death, or censorship if alive at follow‐up date (January 2, 2022), was calculated. Survival analysis was carried out using Cox's proportional‐hazards model. Multivariate survival analysis was performed using a stepwise backward procedure to derive a final model of the variables that had a significant independent relationship with survival. To remove a variable from the model, the corresponding *p*‐value had to be >0.10. Survival curves were plotted using Kaplan–Meier methods and the log‐rank test was applied. All analyses were performed in SPSS Version 27.0 (SPSS Inc).

## 3 RESULTS

### 3.1 Patient characteristics

Data were available for 160 patients (Table 1). Patients were followed up for a median 49.6 (interquartile range [IQR] 31.7–83.2) months. The median age was 67 (IQR 60–72) years and 72% were male, in keeping with reported populations with mRCC. Patients had typical sites of metastases, with lung lesions seen in 108 (68%) patients. Many patients had a single site of metastatic disease (*n* = 86 [54%]), most frequently lung lesions (*n* = 53 [62%]) (Table S1).

**TABLE 1:** Clinical characteristics of patients with metastatic renal cell carcinoma managed with initial active surveillance

Characteristic		*n* (%)	Median (IQR)
Sex	Male	115 (72)	n/a
	Female	45 (38)	
Age	≤64	63 (39)	67 (60–72)
	65–74	70 (44)	
	>74	27 (17)	
ECOG PS	0	90 (56)	n/a
	1	44 (28)	
	2	2 (1)	
	Missing	24 (15)	
Histology type	Clear cell	148 (93)	n/a
	Papillary	9 (6)	
	Other	3 (2)	
Number of metastatic sites	1	86 (54)	n/a
	2	59 (37)	
	≥3	15 (9)	
Sites of metastasis	Adrenal gland	25 (16)	n/a
	Bone	25 (16)	
	Brain/CNS	7 (4)	
	Liver	4 (3)	
	Lung	108 (68)	
	Lymph node	42 (26)	
	Pancreas	12 (8)	
	Peritoneum	3 (2)	
	Renal/renal bed	11 (7)	
	Subcutaneous	4 (3)	
	Other^a^	6 (4)	
Stage at initial diagnosis	I–III	93 (58)	n/a
	IV	67 (42)	
Time from initial diagnosis to metastatic disease	≥1 year	69 (43)	0.7 (0.0–2.7)
	<1 year	91 (57)	
Nephrectomy	Prior curative	93 (58)	n/a
	Cytoreductive	55 (34)	
	None	12 (8)	

^a^ Other included intramuscular, pleura, small bowel, spleen, stomach and thyroid.

Estimated median OS was 88.0 (IQR 34.4–127.0) months (Figure S1). At the time of censoring 82 patients remain alive, for whom minimum and median follow‐up was 19.8 and 59.7 months, respectively.

### 3.2 Nephrectomy status

Ninety‐three (58%) patients presented with recurrent/metastatic disease after prior curative nephrectomy. The median time to metastatic disease in these patients was 26.1 (IQR 11.9–58.6) months, with 24 (26%) developing metastatic disease within 1 year.

Sixty‐seven (42%) patients presented with metastatic disease. Forty‐nine of these underwent cytoreductive nephrectomy within 3 months of diagnosis of mRCC. A further six (9%) had a longer initial period of active surveillance prior to cytoreductive nephrectomy. Amongst patients presenting with metastatic disease, patients who underwent CNx had a longer time to commencing SACT and improved OS compared to those who did not (27.6 (IQR 11.7–82.1) months versus 9.4 (IQR 5.7–17.7) months (*p* < 0.001) and 88.0 (IQR 33.9‐not reached) months versus 29.3 (17.5–48.2) months (*p* < 0.001), respectively).

### 3.3 Metastases‐directed therapy

Twenty‐two (14%) patients underwent elective palliative surgical resection of metastatic disease during their AS period. Contralateral adrenal metastases (*n* = 5) were the most frequently resected metastatic sites (Table S2). Sixteen (73%) patients had oligometastatic disease, and underwent surgery aimed at rendering them free from metastatic disease (Figure S2). Median OS of these patients was 130.3 (89.4‐not reached) months. The remaining six (27%) patients underwent elective palliative metastectomy of a lesion causing significant morbidity, including four patients with symptomatic metastatic brain lesions.

A further six patients underwent palliative radiotherapy to oligoprogressive metastatic lesions involving bone (*n* = 4), lung (*n* = 1) and pancreas (*n* = 1).

### 3.4 tSACT

Median tSACT was 31.8 (IQR 12.0–76.3) months (Figure S3). And 39 patients remain on AS after minimum and median follow‐up of 23.2 and 60.9 months, respectively.

Sixteen patients died prior to commencing SACT. Median survival of these patients was 35.6 (IQR 22.9–64.0) months. Seven died of non‐mRCC related events. Three patients declined SACT following assessment in the specialist clinic. The remaining patients had clinical or radiological evidence of rapidly progressive disease but were no longer of sufficient fitness for SACT. The median survival of these six patients was 51.9 (IQR 30.7–99.8) months.

Given the time period explored, most patients commencing SACT (*n* = 105) received single‐agent VEGFr‐inhibitor therapy (i.e., pazopanib (*n* = 50, 48%), sunitinib (*n* = 31, 30%), tivozanib (*n* = 6, 6%)). Seven patients (7%) commenced ipilimumab plus nivolumab and seven (7%) commenced axitinib plus pembrolizumab. The remaining four (4%) were treated in a clinical trial with pembrolizumab.

### 3.5 Prognostic biomarkers

**FIGURE 1:**
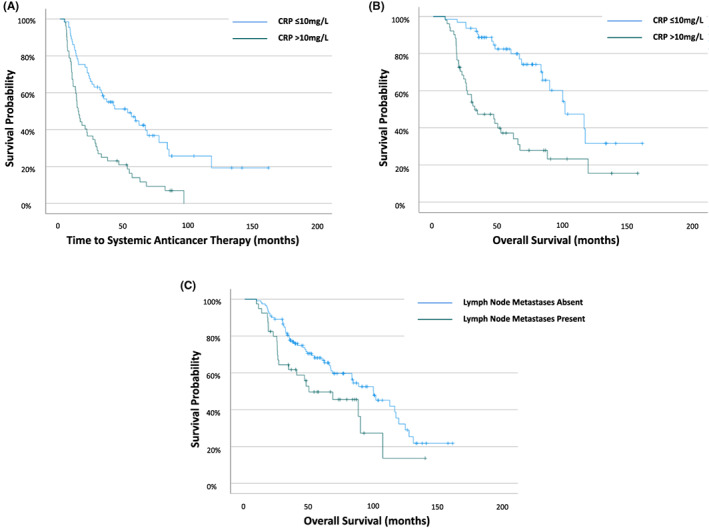
Kaplan–Meier curves examining the relationship between (A) CRP and time to systemic anticancer therapy (tSACT), (B) CRP and overall survival in patients and (C) lymph node metastases and overall survival in patients with metastatic renal cell carcinoma managed with initial active surveillance

The relationship between clinicopathological factors and tSACT was examined (Table 2). On univariate analysis, the number of organs involved (*p* = 0.021), lymph node metastases (*p* = 0.015), histological subtype (*p* < 0.001), c.Ca2+ (*p* < 0.001), albumin (*p* = 0.004), CRP (*p* < 0.001) and mGPS (*p* < 0.001) were predictive of tSACT (Table 3). On multivariate analysis, only CRP (HR 2.47 [95%CI 1.59–3.85] *p* < 0.001) was independently predictive of tSACT. Patients with CRP > 10 mg/L were more likely to commence SACT within 1 year than those with CRP ≤ 10 mg/L (41% vs. 18%, Relative Risk 2.16 [95% CI 1.18–3.96] [*p* = 0.012]). Patients with CRP > 10 mg/L had shorter time to commencing SACT than those with CRP ≤ 10 mg/L (13.8 [IQR 8.5–31.8] months vs. 55.1 [IQR 19.2–84.0] months respectively; *p* < 0.001; Figure 1A).

**TABLE 2:** Prognostic factors at the time of diagnosis of metastatic disease in patients with metastatic renal cell carcinoma managed with initial active surveillance

Characteristic		*n* (%)	Median (IQR)
Haemoglobin (Male)	≥135 g/L	72 (63)	138 (128–147)
	<135 g/L	43 (37)	
Haemoglobin (Female)	≥120 g	34 (76)	130 (121–139)
	<120 g/L	11 (24)	
White cell count	≤11 × 10^9^/L	142 (89)	7.8 (6.4–9.4)
	>11 × 10^9^/L	18 (11)	
Neutrophil Count	≤7.5 × 10^9^/L	136 (85)	5.12 (3.73–6.20)
	>7.5 × 10^9^/L	24 (15)	
Platelet count	≤400 × 10^9^/L	149 (93)	246 (201–310)
	>400 × 10^9^/L	11 (7)	
Corrected calcium	≤2.65 mmol/L	139 (95)	2.41 (2.34–2.48)
	>2.65 mmol/L	7 (5)	
Albumin	<35 g/L	115 (79)	39 (35–42)
	≥35 g/L	31 (21)	
C reactive protein	≤10 mg/L	63 (55)	9 (5–38)
	>10 mg/L	51 (45)	
Modified glasgow prognostic score	0	63 (56)	n/a
	1	27 (24)	
	2	22 (20)	
Fuhrman grade	1–2 (low)	58 (44)	n/a
	3–4 (high)	74 (56)	
Necrosis	Absent	93 (58)	n/a
	Present	67 (42)	
Number IMDC risk factors	0	42 (29)	n/a
	1	58 (36)	
	2	30 (19)	
	3	15 (9)	
	4	1 (1)	
IMDC risk group	Low	42 (29)	n/a
	Intermediate	88 (60)	
	Poor	16 (11)	

Abbreviations: IMDC, International Metastatic RCC Database Consortium; IQR, interquartile range.

**TABLE 3:** The relationship between prognostic factors and time to systemic anticancer therapy or overall survival in patients with metastatic renal cell carcinoma managed with initial active surveillance

	Time to systemic anticancer therapy				Overall Survival			
	Univariate		Multivariate		Univariate		Multivariate	
	HR (95% CI)	*p*	HR (95% CI)	*p*	HR (95% CI)	*p*	HR (95% CI)	*p*
Age (≤64, 65–74, ≥75)	0.82 (0.69–1.074)	0.151			1.24 (0.90–1.71)	0.193		
Sex (male, female)	0.73 (0.49–1.10)	0.130			0.84 (0.51–1.38)	0.484		
Number of organs involved (1, 2, ≥3)	***1.37 (1.05–1.80)***	***0.021***	1.17 (0.86–1.60)	*0.318*	***1.45 (1.04–2.01)***	***0.027***	1.16 (0.78–1.71)	0.463
Lung metastases only (yes, no)	0.80 (0.54–1.18)	0.264			0.78 (0.48–1.27)	0.313		
Lung metastases (absent, present)	1.41 (0.95–2.10)	0.091			1.14 (0.70–1.84)	0.605		
Bone metastases (absent, present)	1.15 (0.71–1.85)	0.58			1.23 (0.70–2.16)	0.480		
Adrenal metastases (absent, present)	1.24 (0.76–2.00)	0.388			1.53 (0.88–2.65)	0.131		
Lymph node metastases (absent, present)	***1.63 (1.10–2.42)***	***0.015***	*1.58 (0.98–2.53)*	*0.061*	***1.92 (1.19–3.10)***	***0.008***	**2.29 (1.27–4.12)**	***0.006***
Histology (ccRCC, non‐ccRCC)	***2.44 (1.48–4.03)***	***<0.001***	1.26 (0.57–2.78)	*0.568*	2.11 (1.00–4.44)	0.050		
Grade (1–2, 3–4)	1.22 (0.83–1.80)	0.316			1.29 (0.80–2.08)	0.297		
Necrosis (absent, present)	1.07 (0.75–1.54)	0.706			0.99 (0.63–1.57)	0.975		
Haemoglobin (male ≥ 135 g/L, <135 g/L: female ≥ 120 g/L, <120 g/L)	0.93 (0.62–1.39)	0.725			1.40 (0.88–2.25)	0.160		
WCC (≤11 × 10^9^/L, >11 × 10^9^/L)	0.91 (0.51–1.62)	0.746			0.95 (0.46–1.98)	0.892		
Neutrophils (≤7.5 × 10^9^/L, >7.5 × 10^9^	0.79 (0.47–1.33)	0.376			0.66 (0.33–1.33)	0.244		
Platelets (≤400 × 10^9^/L, >400 × 10^9^/L)	1.11 (0.54–2.27)	0.786			1.25 (0.54–2.87)	0.605		
Corrected calcium (≤2.65 mmol/L, >2.65 mmol/L)	***4.85 (2.18–10.78)***	***<0.001***	1.99 (0.76–5.22)	*0.165*	1.63 (0.51–5.24)	0.410		
Albumin (≥35 g/L, <35 g/L)	***1.90 (1.22–2.94)***	***0.004***	1.18 (0.68–2.07)	*0.229*	**2.42 (1.44–4.09)**	***0.001***	1.56 (0.84–2.88)	*0.158*
CRP (≤10 mg/L, >10 mg/L)	***2.55 (1.66–3.93)***	***<0.001***	***2.34 (1.49–3.66)***	***<0.001***	**3.40 (1.95–5.91)**	***<0.001***	***3.27 (1.835.86)***	***<0.001***
Modified glasgow prognostic score (0, 1, 2)	***1.64 (1.27–2.11)***	***<0.001***	0.55 (0.18–1.72)	*0.307*	**2.00 (1.47–2.73)**	***<0.001***	0.57 (0.14–2.35)	*0.433*
Time from initial diagnosis to metastatic disease (≤1 year, >1 year)	1.40 (0.97–2.01)	0.074			1.57 (0.98–2.52)	0.061		
Number IMDC risk factors (0–1, >1)	1.18 (0.59–2.13)	0.735			1.14 (0.68–1.91)	0.624		
IMDC group (low, intermediate, poor)	1.21 (0.90–1.64)	0.208			1.31 (0.90–1.93)	0.160		

Abbreviations: HR, hazard ratio; IMDC, International Metastatic RCC Database Consortium; RCC, renal cell carcinoma.

Bold italic values indicate hazard ratio and significant values of *p* < 0.05.

On univariate analysis, number of organs involved (*p* = 0.027), lymph node metastases (*p* = 0.008), albumin (*p* = 0.001), CRP (*p* < 0.001) and mGPS (*p* < 0.001) were predictive of OS (Table 3). On multivariate analysis CRP (HR 3.89 [95%CI 2.15–6.83] *p* < 0.001) and lymph node metastases (HR2.29 [1.27–4.12] *p* = 0.006) were independently predictive of OS. CRP stratified OS from 31.8 (IQR 19.4–87.7) months (CRP > 10 mg/L) to 101.3 (IQR 82.8‐not reached) months (CRP ≤10 mg/L) (*p* < 0.001) (Figure 1B). Median OS was 49.5 (24.7–106.7) months in patients with lymph node metastases versus 99.5 (46.5–130.3) in patients with no evidence of lymph node metastases (*p* = 0.007) (Figure 1C).

The number of IMDC risk factors or IMDC risk group was not predictive of either tSACT or OS in this dataset. A previously proposed prognostic score including a favourable group with no or one IMDC risk factor and two or fewer organs with metastatic disease was also not predictive of tSACT or OS (HR1.28 (95% CI 0.88–1.85), *p* = 0.193 and HR1.39 (95% CI 0.88–2.20), *p* = 0.160, respectively).^4^

Amongst patients presenting with metastatic disease, those who underwent CNx had longer tSACT and OS compared to those who did not 27.6 (IQR 11.7–82.1) months versus 9.4 (IQR 5.7–17.7) months (*p* < 0.001) and 88.0 (IQR 33.9‐not reached) months versus 29.3 (17.5–48.2) months (*p* < 0.001), respectively.

## 4 DISCUSSION

We present the largest cohort of patients with mRCC managed with initial active surveillance and demonstrate reassuring time to SACT and overall survival. For the first time we demonstrate that CRP, a biomarker of systemic inflammation, predicts those patients with a shorter time to requiring SACT and shorter OS.

This study adds to a growing body of evidence that, in carefully selected patients, AS is an appropriate initial management option for patients with mRCC.^4^, ^5^, ^6^, ^7^ Our findings compare well with the only two previously reported studies of AS in this patient group.^4^, ^5^ In a recent real‐world prospective observational study of patients with untreated mRCC, initial AS was pursued in 32% of all patients, with 50% yet to receive SACT after a median follow‐up of 33 (95% CI 29–35) months.^5^ The proportion of patients alive at 3 years was 84% in the AS cohort and 45% in those patients who started SACT soon after diagnosis of mRCC. In a dedicated phase II trial including 48 patients with treatment‐naïve, asymptomatic mRCC, the median time on AS was 14.9 (95% CI 10.6–25.0) months and median OS was 44.5 (95% CI 37.6‐not reached) months.^4^ In our cohort, the median duration of AS was 31.8 months, and median OS was 88.1 months, exceeding that seen in index studies for current standard first‐line SACT options for patients with mRCC.^1^, ^2^, ^17^ Significantly, during the period of AS these patients may benefit from maintained quality of life, with grade 3 or higher adverse events are experienced in up to 75% of patients receiving first‐line SACT.^1^, ^2^

Although this is an observational study, the cohort benefits from being well defined and includes all patients managed by such an approach at a single centre. Significantly, all patients were assessed in the specialist oncology clinic and were suitable for SACT prior to starting AS. Patients were broadly selected for AS if they had low volume, asymptomatic metastatic disease, with physician‐ and patient‐shared decision‐making, in line with current clinical guidelines.^8^, ^9^ There is currently no formal consensus on when to consider therapy in patients undergoing AS. In this study, patients underwent radiological and clinical review in the specialist oncology clinic every 3–4 months initially, as per local guidelines. SACT was typically initiated in those with clinical and/or significant radiological evidence of disease progression or increasing symptomatic burden. In a phase II study of AS in mRCC, approximately half of those meeting the criteria for RECIST‐defined radiological progression continued on AS, highlighting the importance of holistic evaluation of the patient.^4^ We note that a small number of patients (*n* = 6 (4%)) in our cohort died with progressive disease prior to starting SACT. We advocate further work to establish best‐practice follow‐up strategies and criteria for SACT initiation in patients with mRCC managed with initial AS.

Previous studies have suggested a role for IMDC risk factors in patient selection for AS.^4^, ^5^, ^18^ Rini et al identified that the number of IMDC risk factors, either alone or as part of a score in combination with the number of sites of metastatic disease, predicted length of AS.^4^ However, these factors did not predict tSACT or OS in our cohort. Indeed, corrected calcium was the only IMDC risk factor to univariately predict tSACT, but was not associated with OS. Like Harrison et al, we found that 40% of patients in the AS cohort had developed metastases within 1 year of initial diagnosis of RCC, but this factor did not predict tSACT or OS.^5^ The same study also previously demonstrated that patients selected for AS had more favourable IMDC risk scores than those selected for up‐front SACT. However, although IMDC stratified outcomes in those who received immediate SACT, it did not do so in the AS cohort. These findings suggest that patients suitable for AS are a distinct group, with different biological behaviours, compared to those requiring up‐front SACT. This supports the investigation of novel biomarkers of prognosis in this patient group.

Haemoglobin, neutrophil count and platelet count, key parts of the IMDC score, and LDH, a component of the Memorial Sloan‐Kettering Cancer Centre Score for mRCC, are biomarkers of systemic inflammation.^19^ Inflammation plays a key role in the development, survival and progression of cancer and the prognostic significance of circulating blood biomarkers of systemic inflammation, many of which are routinely measured in clinical practice, is well established.^20^, ^21^ In particular, the modified Glasgow Prognostic Score, comprising evaluation of serum albumin and CRP, predicts survival in multiple cancer types.^11^, ^12^ mGPS predicts outcomes in RCC in several clinical settings, including in patients treated curatively with surgery for localised disease and in those with mRCC treated with cytoreductive nephrectomy or cytokine, VEGF‐inhibitor or ICI therapies.^14^, ^22^, ^23^, ^24^, ^25^ To our knowledge it has never been examined in relation to initial AS in patients with mRCC.

In our study mGPS was univariately associated with tSACT and OS, stratifying tSACT from 12.0 (IQR 8.8–13.1) months (mGPS 2) to 14.4 (7.9–53.4) months (mGPS 1) to 67.2 (22.6‐not reached) months (mGPS 0). However, its weighted component, CRP, was the only biomarker explored that predicted tSACT and OS on multivariate analysis. Patients with CRP > 10 mg/L were more likely to start SACT within 1 year and had significantly shorter OS (31.8 vs. 101.3 months (*p* < 0.001)). This simple biomarker of systemic inflammation is cheap, rapidly measured and widely available, and may provide objective evidence to inform clinical decision‐making with patients. For example, upfront SACT may be more appropriate in patients with CRP > 10 mg/L, in whom median tSACT was 13.8 months. Alternatively, these patients may benefit from more frequent clinical and/or radiological assessment during AS, particularly within the first year. Further work is required to define the risks and benefits of these strategies. A limitation of CRP is that, historically, it has not been measured routinely in patients as part of standard pre‐treatment assessment. Indeed, it was only available for 116/160 (71%) of the patients in our cohort. However, CRP measurement has now been incorporated into national UK guidance for minimum datasets in patients with cancer.^26^

We also find that, in this selected cohort, the finding of lymph node metastases is associated with poorer OS, but not tSACT, on multivariate analyses. The prevalence of lymph node metastases was similar in our cohort (26%) to that seen by Rini et al (25%) and Harrison et al (22%).^4^, ^5^ Lymph node metastases are more prevalent in patients with other recognised poor prognostic clinicopathological features, including high Fuhrman grade, tumour size >10 cm and tumour necrosis.^27^ In patients with mRCC metastatic lymph node disease is associated with shorter survival times in patients receiving SACT.^28^, ^29^, ^30^

Our data also highlight the role of surgery in patients with mRCC managed with initial AS. Firstly, the majority (82%) of patients presenting with metastatic disease underwent CNx during their period of AS, typically within the first 3 months. The role of CNx in patients with mRCC is under much debate. To date, studies assessing this strategy have largely focussed on patients requiring upfront SACT.^31^, ^32^ However, several small series have suggested a role for “watch & wait” following CNx in mRCC.^33^, ^34^ In our cohort, amongst patients presenting with metastatic disease, those who underwent CNx had more favourable survival outcomes than the small number of those who did not. These data suggest that, in selected patients, CNx may facilitate a period of initial AS, but further work in the context of patients undergoing CNx is required to understand this. Secondly, 28 (18%) patients underwent local therapy of metastatic lesions, most frequently surgical resection to render them free of metastatic disease. This is similar to the rate of metastases directed therapy observed by Rini et al (17%). The use of metastectomy in patients with mRCC has previously been described, although its exact role remains poorly defined.^35^, ^36^ Approximately 30% of patients undergoing complete resection of metastatic disease remain disease‐free at 5 years.^36^ Harrison et al observed that estimated 3‐year survival was 97% in those with no evidence of metastatic disease and 79% in those with disease present. Our data support this, with median OS 130.3 months in patients rendered free of metastatic disease in our cohort.

Active surveillance is an appropriate initial management option for selected patients with mRCC. These patients avoid the potential adverse effects associated with SACT and demonstrate favourable overall survival. The results of the present study show that CRP, a biomarker of the systemic inflammatory response, may provide additional objective information to assist clinical decision‐making in patients mRCC being considered for initial AS.

## AUTHOR CONTRIBUTIONS

**Mark Stares:** Conceptualization (lead); data curation (lead); formal analysis (lead); investigation (lead); methodology (lead); project administration (lead); resources (equal); supervision (equal); visualization (lead); writing – original draft (lead); writing – review and editing (equal). **Vishwani Chauhan:** Conceptualization (equal); data curation (lead); formal analysis (equal); investigation (lead); methodology (equal); validation (equal); visualization (lead); writing – original draft (lead); writing – review and editing (equal). **Jigi Moudgil‐Joshi:** Data curation (equal); formal analysis (equal); investigation (equal); visualization (supporting); writing – original draft (supporting); writing – review and editing (equal). **Qiu G. Kong:** Data curation (equal); investigation (equal); writing – review and editing (equal). **Jahangeer Malik:** Resources (equal); writing – review and editing (equal). **Aravindhan Sundaramurthy:** Resources (equal); writing – review and editing (equal). **Tony Elliott:** Resources (equal); writing – review and editing (equal). **Edward Mains:** Resources (equal); writing – review and editing (equal). **Steve Leung:** Resources (equal); writing – review and editing (equal). **Alexander Laird:** Methodology (equal); resources (equal); visualization (equal); writing – original draft (equal); writing – review and editing (equal). **Stefan N. Symeonides:** Conceptualization (equal); resources (equal); supervision (equal); visualization (equal); writing – original draft (equal); writing – review and editing (equal).

## FUNDING INFORMATION

This research did not receive any specific grant from funding agencies in the public, commercial, or not‐for‐profit sectors.

## CONFLICT OF INTEREST

Mark Stares, Vishwani Chauhan, Jigi Moudgil‐Joshi, Qiu G. Kong, Jahangeer Malik, Aravindhan Sundaramurthy, Tony Elliott, Edward Mains, and Steve Leung declare no conflicts of interest. Stefan N. Symeonides: Has no personal gain from, or financial interests in, any commercial pharmaceutical entity. Scientific advisory board (institution): BMS, EISAI, EUSA, Merck Serono, MSD, Pfizer. Meetings (institution): BMS, EUSA, Ipsen, MSD. Research funding (institution): MSD. Clinical trials (institution): Eisai, Ipsen, MSD. Educational support: BMS, EUSA, Ipsen, MSD. Alexander Laird: has received speakers fees from BMS.

## Supporting information


Table S1
Table S2
Figure S1
Figure S2
Figure S3
