# Supplementary material for: Initial active surveillance for patients with metastatic renal cell carcinoma: 10 years' experience at a regional cancer Centre
Source: Cancer Med. 2022 Oct 7;12(5):5255–64. doi: 10.1002/cam4.5330 (PMC10028026; doi:10.1002/cam4.5330)
Supplement: Supplementary file 1 — Table S1 Table S2 Figure S1 Figure S2 Figure S3 [file CAM4-12-5255-s001.pdf]

	All (n=160)	Single Site of Disease (n=86)
	**n (%)**	**n (%)**
**Adrenal**	25 (16)	2 (2)
**Bone**	25 (16)	10 (12)
**Brain**	7 (4)	2 (2)
**Liver**	4 (3)	1 (1)
**Lung**	108 (68)	53 (62)
**Pancreas**	12 (8)	4 (6)
**Peritoneal**	3 (2)	2 (2)
**Renal/Renal Bed**	11 (7)	1 (1)
**Subcutaneous**	4 (3)	0 (0)
**Intramuscular**	1 (1)	1 (1)
**Other**	5 (3)	0 (0)
**Lymph Nodes**	42 (26)	10 (12)

Supplementary Table 1: sites of disease at diagnosis of metastatic renal cell carcinoma

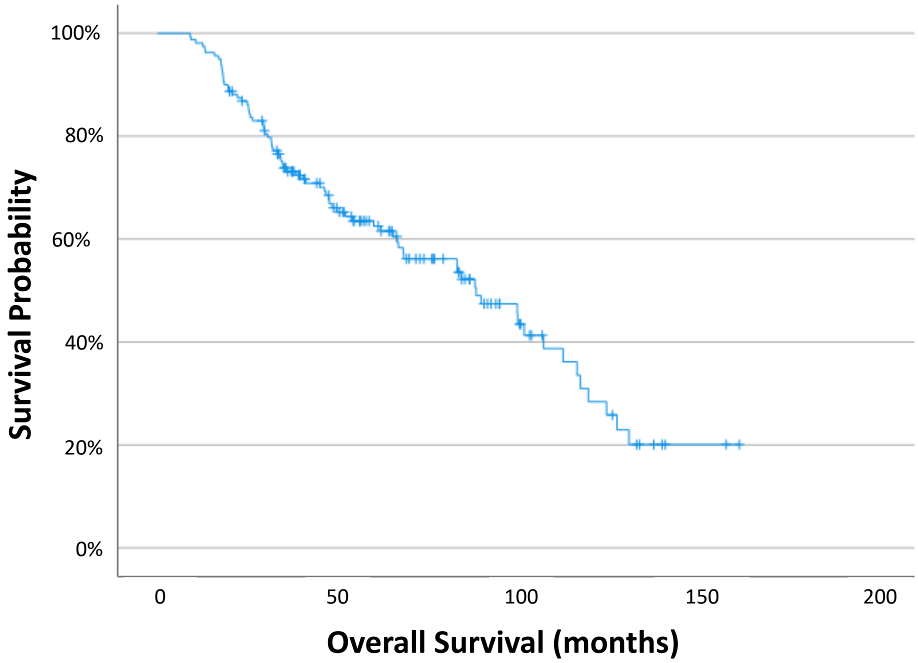

Supplementary Figure 1: Kaplan Meier curve examining overall survival of patients with metastatic renal cell carcinoma managed with initial active surveillance

	All
	**22**
	**n (%)**
**Adrenal**	5 (23)
**Bone**	2 (9)
**Brain**	4 (18)
**Liver**	1 (5)
**Lung**	3 (14)
**Pancreas**	3 (14)
**Other**	3 (14)
**Lymph Nodes**	1 (5)

Supplementary Table 2: Sites of metastatic disease electively resected during active surveillance in patients with metastatic renal cell carcinoma

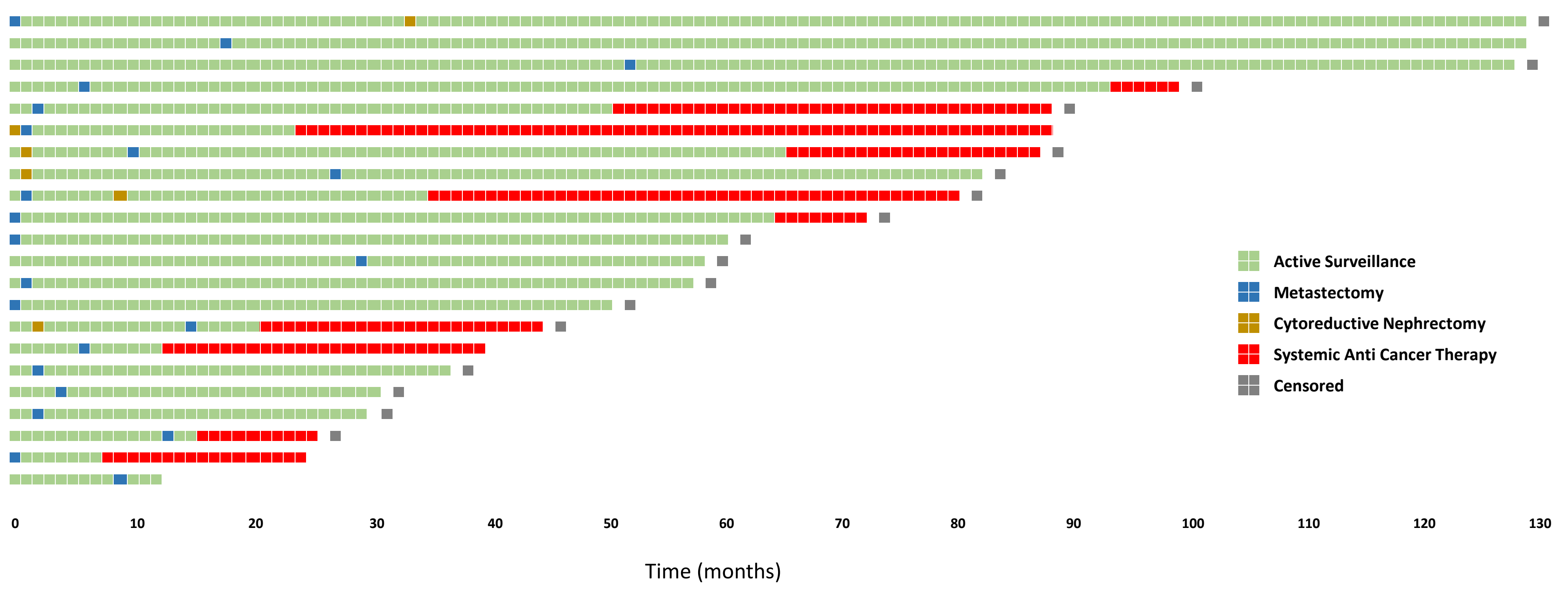

Supplementary Figure 2: Treatment timelines for patients with metastatic renal cell carcinoma initially managed with active surveillance who have undergone metastectomy procedures

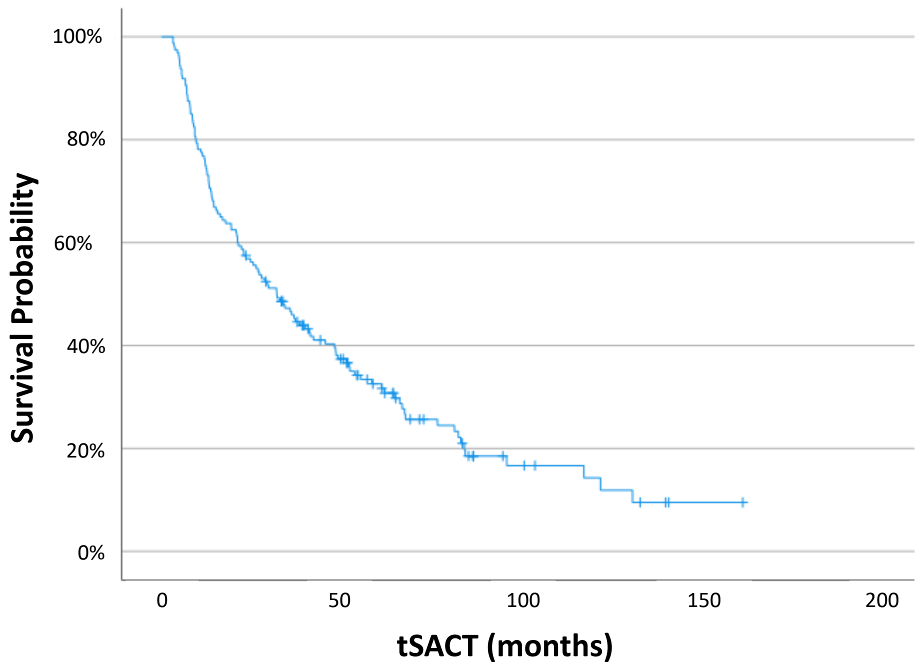

Supplementary Figure 4: Kaplan Meier curve examining time to systemic anticancer therapy in patients with metastatic renal cell carcinoma managed with initial active surveillance
